# The medical AI insurgency: what physicians must know about data to practice with intelligent machines

**DOI:** 10.1038/s41746-019-0138-5

**Published:** 2019-06-28

**Authors:** D. Douglas Miller

**Affiliations:** Medical College of Georgia (GB 3330), Augusta, GA 30912 USA

**Keywords:** Health occupations, Medical ethics

## Abstract

Machine learning (ML) and its parent technology trend, artificial intelligence (AI), are deriving novel insights from ever larger and more complex datasets. Efficient and accurate AI analytics require fastidious data science—the careful curating of knowledge representations in databases, decomposition of data matrices to reduce dimensionality, and preprocessing of datasets to mitigate the confounding effects of messy (i.e., missing, redundant, and outlier) data. Messier, bigger and more dynamic medical datasets create the potential for ML computing systems querying databases to draw erroneous data inferences, portending real-world human health consequences. High-dimensional medical datasets can be static or dynamic. For example, principal component analysis (PCA) used within R computing packages can speed & scale disease association analytics for deriving polygenic risk scores from static gene-expression microarrays. Robust PCA of *k*-dimensional subspace data accelerates image acquisition and reconstruction of dynamic 4-D magnetic resonance imaging studies, enhancing tracking of organ physiology, tissue relaxation parameters, and contrast agent effects. Unlike other data-dense business and scientific sectors, medical AI users must be aware that input data quality limitations can have health implications, potentially reducing analytic model accuracy for predicting clinical disease risks and patient outcomes. As AI technologies find more health applications, physicians should contribute their health domain expertize to rules-/ML-based computer system development, inform input data provenance and recognize the importance of data preprocessing quality assurance *before* interpreting the clinical implications of intelligent machine outputs to patients.

## Introduction

Before the epic sci-fi movie subway fight scene between human hacker Neo and artificial intelligence (AI) being Agent Smith, rebel leader Morpheus observes that Neo is, “beginning to believe” in his power to defeat the Matrix. As forecasted in this 23rd century Machine War, today’s 21st century real-world AI insurgency will also fail without believable data science solutions for high-dimensional data matrix decomposition and signal detection in noisy dynamic (i.e., messy) datasets.

In prior scientific époques, reproducibility of novel research findings was a central tenet of human knowledge expansion.^1^ The inferential statistics of regression and correlation were the analytic gold standard. By disproving the null hypothesis of data similarities, scientists proved the alternatives that pointed to new knowledge. Today, science finds itself at the nexus of quantifiable biology and big data, where knowledge is increasingly represented in immensely complex and rapidly accumulative datasets. In this data-intensive computing era, knowledge acquisition requires new scientific methods. Intelligent machines use discriminative modeling to learn features hidden within data manifolds, revealing insights otherwise obscure to humans. A key to extracting such unobvious commonalities from complex high-dimensional datasets is robust data dimensionality reduction and meticulous matrix decomposition.

This new data-intensive scientific era raises many questions. What is the right data (i.e., size, source, and quality) upon which to train a learning computer system? Can humans unknowingly (or intentionally) curate datasets that cause machines to confidently predict erroneous outcomes? Can ever messier, bigger and more dynamic data thwart intelligent machines? It is well-known that answering these crunchy technology questions requires both data and computing scientists to be well-informed on the exquisite interdependencies between machine intelligence, knowledge representation, and dynamic data. And for human health AI applications, do input data quality and provenance issues pose unique health risks that demand earlier and more meaningful involvement of medical domain experts?

What has gone largely unrecognized by AI developers and potential users is that the medical applications of machine intelligence are highly susceptible to proper handling of increasingly large, messy, and dynamic data inputs,^2,3^ and that medical data matrix glitches can indeed have human health consequences. Broadly framed by data science and AI computing fundamentals, this perspective offers focused insights into the challenges health professionals face when translating high technologies to the human condition.

### Machine intelligence

Computer scientist and AI guru Andrew Ng (of Google Brain, Baidu, and NVDIA) has offered the view that, “The measure of a good AI technology is that it does well what humans can do easily in one second”.^4^ While a machine that truly mimics higher cognitive function awaits human design,^5^ AI technologies are accelerating complex problem-solving in data-dense sectors like finance, cyber-security, social media, econometrics, computer vision, and logistics tracking (i.e., blockchain).^6,7^

Discriminative models for supervised machine learning (ML) are typically programmed to predict how an exploratory testing dataset relates to trusted training data.^7^ Preprocessing of training datasets enhances the yield of AI analytic modules for reliably selecting the most crucial features and faults, potentially rendering advanced AI modules automated (i.e., unsupervised deep learning (DL)).^7^ Unlike humans who can effectively transfer past experiences and expertise to new tasks, AI modules that generalize poorly to new datasets (other than those it trained on) can cause massive ML failures.^8^ Careful preprocessing of exploratory testing datasets before AI analytics helps to generalize knowledge in subsequent testing dataset runs.

Computer programming and DL expert François Chollet (of Google AI and Keras) attributes AI’s recent remarkable success as follows, “You can achieve a surprising amount using only a small set of very basic techniques”.^9^ Pure DL engines are discriminative modeling algorithms, systems of neural networks (i.e., nets) with multiple hidden layers in which neurons receive, weigh and combine inputs to produce an output that is passed to the next layer.^7^ Such relative simplicity implies that one need not be an AI technology expert to accomplish DL. But such simplicity also belies the fact that the inherent data structure is often the missing link to efficient and accurate AI analytics.

Innately different data structures (i.e., text, images, telemetry signals, and video) demand different computational approaches. For example, imaging data structures (i.e., natural or digital image pixel arrays) are best learned using convolutional neural nets, while linguistic text structures (i.e., natural language interfaces) are better learned by recurrent neural nets. Absent clear neural net comprehension of these innate data structures, even the fastest graphics processing units, and most massive data troves are not very good learners.

And not all datasets can be trusted. Very slight data matrix perturbations, introduced intentionally into discriminative neural nets by generative adversarial nets (GAN’s), can cause an AI module to become 99% certain of a predictive model output that human experts immediately recognize as 100% erroneous.^10^ The dual goals of purposefully pitting generative nets against discriminative nets are better discriminator object and feature identification (i.e., reinforcement learning), and better generator learning about how to deceive discriminators. Such net-versus-net rivalry instills machines with artificial imagination, a creativity that can contribute to novel in silico prodrug design.^11^ Google AI chief scientist Yann LeCun calls GAN’s, “The coolest idea in DL in the last 20 years”.^12^ However, uncurated or poorly preprocessed original training datasets have a chilling effect on GAN performance.

### Knowledge representation

Many organizations share a common problem—they have a lot of data, but their data structure does not match a particular question that they want to solve. For AI start-ups and businesses outside “The Big Nine” (i.e., Alibaba, Amazon, Apple, Baidu, Facebook, Google, IBM, Microsoft, and Tencent), creating a curated high-quality training dataset is not easy.^13^ As cognitive computing and big data analytics innovator Adrian Bowles (of IBM, GTE, and Aragon Research) has opined, “There is no machine intelligence without knowledge representation.”^14^ So knowledge (i.e., digits, facts, beliefs, pictures, and general information) must be placed within context for machines to test the validity of what they have learned against other existing or new inputs. Without a clear data map, intelligent machines cannot make sense of data inputs.

Data scientists traditionally spend 70–80% of their time cleaning data and choosing the right data to mine.^15^ Clear data taxonomies provide ordered representations of the formal structure of knowledge classes or types of objects within a data domain. Ontologies use data rules and representations to factor in how object types relate to each other, and how data domains influence objects within them. Taxonomies and ontologies help ordered proximity algorithms (i.e., k-word nearest neighbor search for text and fast nearest neighbor search for dynamic indexing) make statistical inferences and associations based on geometric distance functions (i.e., vector calculus) that reflect data similarity, or dissimilarity.^16,17^ More advanced analytics (like AI) compel data scientists to create tools and technologies that can wrangle an ever-expanding and more complex data universe^2,3^—cleaning, labeling, organizing, and integrating data from many different sources.^18^

Computer languages for querying databases (i.e., C++, Java, and C#) follow set data rules and programming methods. Static methods depend on data class; they are object-oriented, requiring objects to access variables inside of a data class (i.e., static or class variables). Nonstatic methods require an instance be created (i.e., instantiated); they access the individual characteristics (i.e., nonstatic or instance variables) of each object being generated from the data class. *R* is one statistical computing and software platform comprised of functions, data and compiled code in well-defined formats. Open-access *R* platforms enable users to carry out advanced statistical operations (i.e., data aggregation, cleaning, analysis, and representation) and data mining (i.e., time-series forecasting, data visualization, string manipulation, and predictive modeling) in diverse data spaces.^18^

Rules-based computing systems make inferences from data. Their data-driven static analyses (with Java, Python or R) use large amounts of code to infer coding rules to guide a good data analysis strategy. Given a set of facts (i.e., a knowledge base) and a set of rules (i.e., “if-then” coding statements), a rules-based system directs the computer to a recommendation. Human experts in the knowledge domain can help to specify the steps to making a decision and can identify special cases (i.e., expert-based rules). By comparison, ML-based computing systems often begin by searching large, heterogeneous and complex data spaces (i.e., query-database communication).^19^ ML outputs identified by historical training data runs are derived from a combination of numerous input variables, and modeled into learned patterns (i.e., features). Unlike rules-based systems, ML systems rely only on the outcomes knowledge of experts in the problem domain, and ML rules are largely inferred by feature engineering, with the goal of predicting a future outcome (i.e., causal inference).^7,20^ Analogous to human reasoning, ML is more adaptive to continuously improved data preparation.

### Dynamic data

“Many mathematical objects can be understood better by breaking them into constituent parts, or finding some properties of them that are universal, not caused by the way we choose to represent them”.

This quote from leading AI scientist, Yoshua Bengio (of Université de Montréal), reflects the fact that many of today’s AI computing breakthroughs are predicated on data science solutions.^21^ Increasingly, modern datasets are dynamic, arriving sequentially in batches over time that are potentially correlated (i.e., time-series data) and stored in multiple locations (i.e., distributed data networks). Data scientists know that these dynamic high-dimensional datasets are often messy—corrupted by redundant data, outliers and missing entries requiring robust preanalysis data completion and/or data recovery.^15^

Dr. Bengio continues, “We can also decompose matrices in ways that show us information about their functional properties that is not obvious from the representation of the matrix as an array of elements.”

Data matrix decomposition simplifies the AI knowledge extraction process, reducing computation time and minimizing the risk of predictive model over-fitting. In traditional multivariate datasets, the number of features or variables being collected (data dimensions, *p*) is smaller than the number of cases (data elements, *n*). The recent explosion of dynamic data has spawned a high-dimensional data era wherein *p* is often orders of magnitude greater than *n*.^22^ AI’s full future impact is largely predicated on high-dimensional data reduction and signal detection in noisy dynamic datasets.

Computing run times of simple AI diagnostic modules (i.e., auto-encoders and support vector machines), advanced convolutional or recurrent neural networks, and other DL algorithms depend greatly on the dimensionality of the inputted data. To achieve computing efficiencies, data scientists use a wide variety of dimensionality reduction and feature selection techniques: principal component analysis (PCA),^2^ generalized spike models,^22^ robust PCA,^23,24^ PCA whitening,^25^ robust subspace tracking,^24^ low rank plus sparse [L + S] data decomposition^26^ and algorithms (i.e., *t*-distributed stochastic neighbor embedding).^27^ With proper preprocessing of dynamic datasets, AI technologies are becoming more efficient at signal processing (i.e., satellite communications and seismology), computer vision (i.e., video surveillance and traffic patterns), and network traffic analysis.

### Entering the medical matrix

Binary numeric (i.e., digital) datasets are ideal for training neural net algorithms for (un-)supervised feature recognition in complex data matrices. Digital medical images representing human anatomical structures and physiological functions are large (i.e., magnetic resonance imaging (MRI) = 200 MB per image), but generally clean data files.^28,29^ This partly explains why applying AI technologies to digital medical imaging (i.e., radiology, dermatology, histopathology, and retinal photography) datasets translates well, achieving ≥95% of human accuracy for predicting disease types and severity.^6^ But while digital medical imaging data quality is often superior to other medical data, it too can be messy. As such, attention must be paid to the careful preprocessing of all medical datasets, whether the data are static or dynamic.

Static Datasets—once utilized to distinguish AM from FM radio signals, PCA vector calculus is now used to de-convolute digital electrocardiogram^30^ and electroencephalogram^31^ recording signals. When applied to static body imaging, the generalized spike model detects localized disease-induced anatomical variations of organ landmarks wherein the PCA eigenvectors are rendered sparse.^22^ Robust PCA of static brain MRI studies can separate clinically useful functional from diffusion MRI information.^23^

PCA preprocessing of genomic data (by creating file-backed big matrix objects for analyses using R package algorithms and statistical tools) defines relevant gene expression clusters within high-dimensional genomic data arrays, rapidly creating polygenic risk scores for conditions like celiac disease.^32^ Conventional PCA and hybrid ML algorithm analyses of heterogeneous high-dimensional (i.e., neuroimaging and biomarkers) and low-dimensional (i.e., medical records) data manifolds have shown comparable (83–85%) accuracy for discriminating healthy subjects from those with mild cognitive impairment and patients with documented Alzheimer’s disease.^33^

**Fig. 1:**
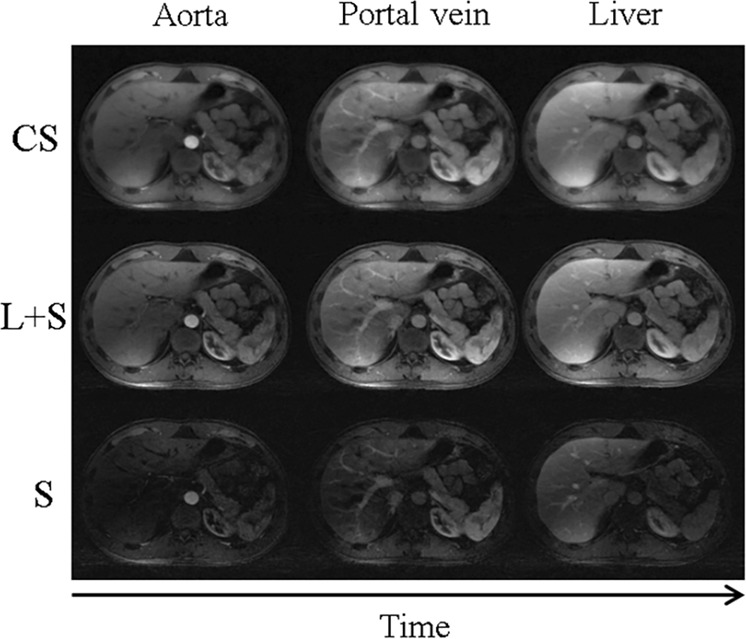
Data matrix decomposition for accelerated dynamic MRI of three contrast enhanced image phases.^18^ Compressed sensing (CS) images reflect sparsity only, while low rank plus sparse (L + S) images provide improved spatiotemporal resolution resulting from the automated background suppression of the sparse (S) components, enhancing contrast resolution. (Reproduced with the permission of the Institute of Electrical and Electronics Engineers)

**Fig. 2:**
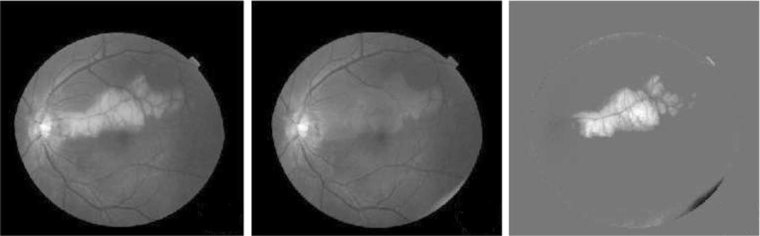
Change detection method of robust principal component analysis (PCA) combined with low rank plus sparse (L + S) matrix decomposition.^18^ The original image (left), low-rank clean component (center), and sparse component image (right) reflect disease progression primarily localized to the retinal fundus.^28^ (Reproduced with the permission of the Institute of Electrical and Electronics Engineers)

Applying low rank plus sparse (L + S) decomposition to contrast-enhanced digital subtraction imaging allows for automated background subtraction of S component differences (i.e., perturbations), while L images are useful for image-to-image realignment and change detection^23^ (Fig. 1). RPCA-based change detection methods reflecting S component perturbations between an original image and the L image dataset can demonstrate spatiotemporal disease progression (i.e., angiography of vascular diseases and^23^ fundoscopy of retinal diseases^34^) (Fig. 2).

Dynamic Datasets—Encoding of clinically relevant spatiotemporal information in dynamic MRI datasets—organ motion, contrast agent uptake, tissue signal relaxation, etc.—requires data acquisition at each gated time point to be faster than the physiological process being studied. MRI hardware is too slow to fully sample data during dynamic 4-D imaging. RPCA is helpful for detecting sparse outliers in the under-sampled *k*-dimensional subspace within dynamic MRI datasets, and is useful for region-of-interest detection and tracking.^23^

Fully cinematic MRI data acquisition at each time point is inefficient because of information redundancy across multiple image frames. By learning inter-frame motion fields, RPCA improves cine MRI resolution without creating aliasing artifacts, and accelerates image reconstruction via background noise suppression. RPCA spatiotemporal data correlations at each time point permit reconstruction of time-series datasets of related dynamic MRI images; the resulting near-instantaneous data snapshots increase acquisition speeds.^26^

### Matrix medical complications

The blending of statistical methods with computer science must take human utilities into account when designing inference and decision-making systems. This is of particular importance in the medicine, where the application of AI technologies with the intention of caring for others without robust consideration of medical dataset characteristics can predispose unique human health complications.

The first complication-predisposing condition is analytic system reliability, which is fundamentally related to AI module design. AI modules are trained on datasets and purposefully engineered to *not* achieve 100% predictive accuracy for feature detection, in order to be generalizable for learning on other new testing datasets. Such inherent AI module mutations can have health application consequences. Although diagnostic accuracies ≥95% are achieved using ML or DL analytics of relatively clean, static digital medical imaging datasets, this high accuracy level is not reproducible when using messy, dynamic data inputs.^6,29^ Dataset preparation should be informed by medical experts, with appropriate business due-diligence to avoid financial pressures on the dataset preparers.

The second risk comes when interpreting AI output data to patients in the clinical setting. Medical testing discussions are often fraught due to physician–patient knowledge imbalances, especially when the diagnostics and related care options are complex. Doctors cannot ethically relate AI model results for predicting an important outcome (i.e., the genetic odds of disease^32^ or likelihood of inpatient death^35^) to their patients without also plausibly explaining how the “black box” generated those odds.^6,36^ While doctors might assume that the precision of an approved AI medical applications is high, doctors disintermediated from the training data cannot vouch for either the quality of the raw data or the rigor of data preprocessing.^28^

Thirdly, while data scientists know that data provenance—the origin of datasets in time and place—is a key determinant of the inferences to be drawn from it, most physicians do not. Fetal ultrasound markers of Downs Syndrome derived using 1990s low-res 2-D ultrasound source data do not carry the same predictive value when remodeled with modern high-res 3-D imaging fetal ultrasound datasets.^37^ And while ML analytics of 3-D fetal ultrasound imaging data at scale could augment diagnostic observer reproducibility, it could also skew individual case medical decision-making as compared to human experts (i.e., the need for amniocentesis to detect the trisomy 21 chromosome). When Google AI’s Automated Retinal Disease Assessment tool was field tested in India’s rural population,^38^ inferences drawn using cohort training datasets from developed world places could not be readily translated in the undeveloped world.

### The AI insurgency—learning from the matrix revolutions

In the Matrix movie trilogy, the Oracle’s revolutionary cause was to save humanity by unbalancing algorithms. When first meeting Neo, her human instrument of digital disruption, the Oracle points to a sign bearing the words of Socrates—*Temet Nosce* (Know Thyself). Modern physicians know that the grounding premise of medical practice remains scientific knowledge. However, the undisciplined pursuit of neo-technologies by AI-enthused medical users in the absence of transparent input data quality assurances could unknowingly do harm in clinical practice.

Techniques long applied for data matrix decomposition in computer science and engineering are now being used to wrangle high-dimensional dynamic medical datasets. Physicians wanting to put AI into meaningful use in clinical practice need not be data or AI experts. But in today’s rapidly evolving data-intensive AI insurgency, if (like Neo) health professionals freely choose to enter the Matrix, then (like the Oracle) they must deeply understand and reflect on the human health impacts of knowledge representation and dynamic data on machine intelligence.

When Neo asks, “What is the Matrix?”, Morpheus responds, “Control”. Modern medicine has entered the Matrix. Once inside, health professionals must proceed deliberately, endeavoring to first grasp the limits of such powerful AI technologies before embracing them. Not unlike CGI battle adversaries Neo and Agent Smith, nonadversarial networking of medical, data and computing experts could reveal critical strengths and weaknesses of rival scientific methods. By engaging to jointly inform the inferences derived from complex medical datasets, these AI insurgents could derive deep understanding from data obscurity, coming to “believe” in their capacity to translate AI technologies into improved patient care.

To do less would cede control to the matrix.
